# Effectiveness and Adverse Effect of Intravenous Lacosamide in Nonconvulsive Status Epilepticus and Acute Repetitive Seizures in Children

**DOI:** 10.1155/2018/8432859

**Published:** 2018-06-10

**Authors:** Monsicha Ngampoopun, Piradee Suwanpakdee, Nattapon Jaisupa, Charcrin Nabangchang

**Affiliations:** ^1^Neurology Division, Department of Pediatrics, Phramongkutklao Hospital, Bangkok, Thailand; ^2^Department of Pharmacology, Phramongkutklao College of Medicine, Bangkok, Thailand

## Abstract

Nonconvulsive status epilepticus (NCSE) and acute repetitive seizures (ARS) are associated with significant morbidity and mortality. Due to the lack of randomized-controlled trials of intravenous antiepileptic drugs (AEDs) in these conditions, trials of a new generation of AEDs in this aspect are needed. A prospective interventional study was conducted in children under 18 years of age with NCSE or ARS who either had contraindication to or were refractory to first-line AEDs and received intravenous lacosamide. Demographic data, the efficacy of treatment, and adverse effects were recorded. Eleven patients with a median age of 11 years, predominantly female (72.7%), were enrolled. Average loading dose was 227 mg (8.3 mg/kg/dose) and average daily maintenance dose was 249 mg (4.6 mg/kg/dose). All patients (100%) experienced a reduction in seizure frequency within 24 hours. Eight of eleven patients (72.7%) experienced a reduction in seizure frequency of more than 50% by the end of the study, and one patient became seizure-free. In terms of adverse events, one patient had a bradycardia without prolongation of the PR interval. Interestingly, there was a case of neuronal ceroid lipofuscinosis in which a significant improvement in seizure control was achieved. The results indicate that intravenous lacosamide may be an alternative treatment for NCSE or ARS in children. To our knowledge, this is the first study on the use of intravenous lacosamide in Asian children. This study is registered to Thai Clinical Trials Registry (TCTR) and the trial registration number is TCTR20180508004.

## 1. Introduction

Nonconvulsive status epilepticus (NCSE) and acute repetitive seizures (ARS) are associated with significant morbidity and mortality and require prompt and effective treatment. Benzodiazepine, phenytoin, valproic acid, and phenobarbital are the first-line antiepileptic drugs for status epilepticus (SE) and ARS. If the seizure is refractory to the first-line treatment, the use of high dose barbiturates, propofol, or midazolam is recommended [1]. Adverse effects of these substances on consciousness and respiration may require ICU admission and artificial ventilation. Therefore, drugs with better adverse effect profiles may be beneficial for patients [2].

Lacosamide (LCM) is a novel agent that has been approved by the US Food and Drug Administration for individuals aged 4 years and older for partial-onset seizures as monotherapy or adjunctive therapy. Intravenous (i.v.) LCM has safety and tolerability similar to that of oral LCM [3]. Lacosamide has a novel dual mode of action. First, it has a functionalized amino acid that selectively enhances slow inactivation of voltage-gated sodium channels, increasing the proportion of sodium channels unavailable for depolarization. Such unavailability reduces pathological hyperexcitability without affecting normal physiological activity of neurons. Second, LCM binds to collapsin response mediator protein-2 (CRMP2), which enhances the drug's antiepileptic activity. However, this mechanism has recently been questioned [4].

Lacosamide is eliminated by the kidneys and has not been shown to affect CYP enzymes or biotransformation in the liver. It has a half-life of approximately 13 hours and is minimally (less than 15%) bound to serum proteins [4]. It is relatively well-tolerated. Common side effects include dizziness, headache, nausea, vertigo, somnolence, ataxia, and, in a small number of patients, a prolongation of the PR interval and atrioventricular block on electrocardiography [5, 6].

Many clinical studies had shown that LCM has a good response rate in adults with epilepsy and has a favorable side-effect profile [7, 8]. However, there is limited data on the efficacy of intravenous LCM in the pediatric population, especially in Asian children [9–12]. Therefore, a single center, prospective interventional study of the efficacy and tolerability of intravenous LCM as adjunctive treatment or monotherapy in Thai children aged less than 18 years with nonconvulsive status epilepticus or acute repetitive seizures was conducted.

## 2. Patients and Methods

This single center, prospective, interventional, open-label study was conducted between April 2016 and March 2018 in the Phramongkutklao Hospital, Thailand. The study protocol, amendments, and informed consent were approved by the Institutional Review Board of the Royal Thai Army Medical Department. All patients or their legal representatives signed written informed consent before the participation in the study.

Patients were selected based on the following criteria: (i) patients with status epilepticus and/or acute repetitive seizures aged less than 18 years; (ii) patients who had uncontrollable seizures after first-line antiepileptic drug therapies; and (iii) patients with a contraindication to first-line antiepileptic drugs (allergy to drugs, comorbidity, drug interaction, and risk of side effects). The electrocardiogram and tests of kidney and liver function were done before the enrollment to rule out atrioventricular heart block and severe liver and renal diseases. Lacosamide therapy would be terminated if a patient experienced intolerable side effects or seizure aggravation.

For the purposes of this study, acute repetitive seizures (ARS) were defined as two or more seizures in 24 hours, with self-limited seizures, and patients resuming their normal state after each seizure. Convulsive status epilepticus (CSE) was defined as continuous convulsive seizures lasting more than 5 min or two or more seizures during which the patient did not return to baseline consciousness. Nonconvulsive status epilepticus (NCSE) was defined as a change in mental status from baseline for more than 30 min with ictal discharge on the electroencephalogram (EEG) [2].

The data and clinical findings were recorded including sex, age, etiology, epilepsy history, seizure type, onset of seizure, order in which AEDs were administered, loading and maintenance doses of intravenous LCM, and concomitant AEDs, as well as the responsiveness to the LCM therapy and adverse events.

Intravenous LCM was added to the medications administered as a part of a standard protocol, including a sequence of benzodiazepine, phenytoin, valproic acid, and/or phenobarbital. Thus the seizures in these patients were refractory to conventional treatments. The starting loading dose of LCM was 10 mg/kg/dose (maximal dose of 400 mg/dose) followed by the maintenance dose of 1-10 mg/kg/day which was administered twice per day for three days. LCM was given orally after the discontinuation of LCM i.v. Cessation of seizures was defined as the disappearance of EEG seizure activity (all patients with a diagnosis of nonconvulsive status epilepticus underwent continuous EEG monitoring) or the disappearance of previous ictal symptoms without any suspicion of ongoing subclinical seizure.

The response to the treatment was defined by a comparison of baseline seizure frequency 1 month prior to the study to the frequency during i.v. LCM treatment at 24 hours and 1 week. It was classified as seizure-free, >75% reduction, >50% reduction in seizures, and ineffectiveness (all patients with less than 50% reduction). Children and adolescents with more than 50% reduction in seizure frequency during a minimum period of 1 week were considered responders. Wilcoxon rank test was used for statistical comparison between seizure frequency 1 month before the study and after using LCM i.v. for 24 hours and 1 week. Immediate side effects within 48 hours and short-term side effects (within 1 week) of i.v. LCM administration were also noted.

## 3. Results

Twelve patients met the inclusion criteria, but one was excluded from the study due to a noncompliance. Therefore, eleven patients were included in the study. Nine patients (81.8%) had acute repetitive seizures, and two (18.2%) had nonconvulsive status epilepticus. The demographic data are shown in the Table 1. The patients were aged 7-16 years (median age 11 years) and were predominantly female (72.7%). Most patients (90.9%) had underlying preexisting epilepsy including focal epilepsy of unknown etiology (36.3%) and Lennox-Gastaut syndrome (27.2%) and had received a median of 3.5 AEDs (range 2-5) concomitantly. The mean age at seizure onset was 4.4 ± 4.3 years. Lacosamide was administered as the second-order and third-order antiseizure medication in 5 of 11 (45.4%) and 4 of 11 (36.4%) patients, respectively.

**Figure 1:**
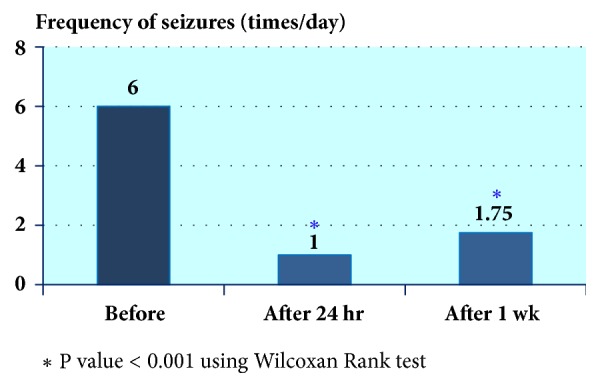
Comparison between seizure baseline 1 month before IV LCM and after IV LCM at 24 hours and 1 week.

The average loading dose was 227 mg (8.3 mg/kg/dose) and average maintenance dose was 125 mg, prescribed twice per day (4.6 mg/kg/dose). Intravenous LCM was found to be efficacious in every patient (100%) with a reduction in seizure frequency within 24 hours of administration. Eight patients (72.7%) were considered responders at the end of the study, and one of them became seizure-free. There was statistically significant reduction in seizure frequency after i.v. LCM (p < 0.05; Figure 1.)

In terms of adverse effects, one patient experienced bradycardia without the prolongation of the PR interval. No other adverse effects and no hemodynamic instability during the infusion were documented in this study. Interestingly, there was one patient who was diagnosed with neuronal ceroid lipofuscinosis (NCL), which is generally refractory to other AEDs; this patient showed significant improvement in seizure frequency as described below.

### 3.1. Patient Data

A 9-year-old boy with a history of neuronal ceroid lipofuscinosis with acute repetitive seizures, in bedridden status, was admitted to the Phramongkutklao Hospital due to community-acquired pneumonia and had myoclonic seizures more than 100 times per day. Despite antiepileptic treatment which included topiramate 10 mg/kg/day, clonazepam 6 mg/kg/day, levetiracetam 40 mg/kg/day, valproic acid 60 mg/kg/day, and lamotrigine 6 mg/kg/day, the epilepsy was poorly controlled. At admission, intravenous benzodiazepine was started, followed by intravenous levetiracetam at 30 mg/kg/dose. Despite the treatment, the patient continued to have ARS, and therefore intravenous LCM was initiated (loading dose 8 mg/kg/dose followed by maintenance dose 5 mg/kg/dose twice per day). The patient experienced an 85% decrease in seizure frequency in 24 hours and a 70% decrease by the end of the study.

## 4. Discussion

Intravenous LCM is known to be an effective and well-tolerated treatment for SE and ARS in hospitalized adult patients [13]. Currently, there are only limited data from retrospective trials in children. Arkilo et al. published a retrospective study of 47 pediatric patients who received intravenous LCM. The initial dose ranged from 2 to 10 mg/kg, with the effectiveness of 65%. Sedation was noted in 5 children without any other identified adverse events [14]. Grosso et al. published a retrospective case study of 11 pediatric patients with status epilepticus who were administered intravenous LCM as third or higher line of antiseizure medications. Seizure cessation was observed in 45% of patients, with no identified serious adverse effects with high loading dose up to 14 mg/kg/day [15]. Poddar et al. published a retrospective study of 9 pediatric patients receiving intravenous LCM for the treatment of status epilepticus. In this study, the success rate was 77.8%, and 44.4% of patients became seizure-free. Better outcomes were observed when LCM i.v. was given earlier with adequate dosing. The mean initial loading dose was 8.7 mg/kg. Bradycardia occurred in one patient within 24 hours after initiating LCM, but no other adverse effects were reported [16]. Sample sizes in our study are similar to those in previously published studies, and our results support earlier findings regarding the efficacy and adverse effect profile of LCM. No significant adverse reactions or drug-drug interactions were observed. One patient from our cohort had transient bradycardia, which resolved without intervention, and the patient was hemodynamically stable. Extending previous reports, our study also demonstrated statistically significant reduction in seizure frequency at 24 hours and 1 week after initiation of i.v. treatment with LCM.

There is a recent study on the possible association between LCM treatment and a change in CRMP2 function [17]. The exact contribution of this reorganization to epileptogenesis is not yet fully understood. In a mouse model, CRMP2 has been associated with neurodegenerative diseases including NCLs [18]. Due to this association, LCM may be a therapeutic option for NCLs or other neurodegenerative diseases, but available clinical data is limited. Interestingly, our study included one patient with the NCL who had favorable outcome in seizure frequency. We followed up with this patient every week for two months. His mother reported that the seizure frequency decreased more than 50% while the patient continued to take LCM orally. Therefore, our observation can provide a foundation for future use of LCM as an antiepileptic drug for the treatment of this particular disease.

The strength of our study is its prospective design and statistical significance in seizure cessation. However, the limitations include a small sample size, the variability in concomitant antiepileptic drugs, and the absence of patients with convulsive status epilepticus. Therefore, a larger prospective clinical study with more types of seizures needs to be conducted to establish the efficacy of i.v. LCM in SE or ARS in children.

## 5. Conclusions

Our prospective study demonstrated that intravenous LCM is safe and efficacious in NCSE or ARS in children. Our findings suggest that i.v. LCM can be a good alternative treatment before considering anesthetic agents, especially when ICU bed availability is limited.

## Figures and Tables

**Table 1:** 

Patient	Age/sex	Etiology	Other AEDs	Order of IV LCM	Loading/maintenance dose (MKDose)	% seizure reduction in 24 hr	Efficacy (% seizure reduction)	Side effect
***1***	*10 Y*	*Traumatic brain injury*	*LEV, CZP, VPA, TPM*	*3*	*10/6.6*	*85.71*	*No change*	*No*
***2***	*12 M*	*Lennox-Gastaut syndrome*	*LEV, DZP, CLB, TPM, PPN*	*2*	*10/5*	*75*	*>50%*	*bradycardia*
***3***	*15 Y*	*Anti NMDAR encephalitis*	*LEV, TPM*	*1*	*6.7/2.2*	*100*	*Seizure free*	*No*
***4***	*9 M*	*neuronal ceroid lipofusinosis*	*LEV, CZP, VPA, TPM, LTG*	*3*	*8/5*	*85*	*>50%*	*No*
***5***	*9 Y*	*focal epilepsy of unknown etiology *	*LEV, PHT*	*4*	*4.7/4.7*	*100*	*No change*	*No*
***6***	*14 Y*	*focal epilepsy of unknown etiology *	*LEV, VPA, TPM*	*3*	*5/2.5*	*100*	*>50%*	*No*
***7***	*14 Y*	*Lennox-Gastaut syndrome*	*LEV, TPM, CLB, PB*	*2*	*9/4.5*	*90*	*>75%*	*No*
***8***	*10 Y*	*focal epilepsy of unknown etiology*	*VPA, TPM, CBZ*	*2*	*9/4.5*	*100*	*>75%*	*No*
***9***	*7 Y*	*Schizencephaly*	*TPM, DZP, PB*	*3*	*10/5*	*66.67*	*>75%*	*No*
***10***	*16 Y*	*focal epilepsy of unknown etiology*	*PHT, TPM, CZP*	*2*	*10/5*	*100*	*No change*	*No*
***11***	*8 M*	*Lennox-Gastaut syndrome*	*LEV, PPN, LTG, PB*	*2*	*8.7/5.2*	*71.42*	*>75%*	*No*

AEDs: antiepileptic drugs; IV: Intravenous; LCM: lacosamide; LEV: levetiracetam; CZP: clonazepam; VPA: valproic acid; TPM: topiramate; DZP: diazepam; CLB: clobazam; PPN: perampanel; LTG: lamotrigine; PHT: phenytoin; PB: phenobarbital; CBZ: carbamazepine.

## Data Availability

The datasets generated during and/or analysed during the study are available from the corresponding author on reasonable request.

## Abbreviations

SE: Status epilepticus
NCSE: Nonconvulsive status epilepticus
ARS: Acute repetitive seizures
NCL: Neuronal ceroid lipofuscinosis
AEDs: Antiepileptic drugs
IV: Intravenous
LCM: Lacosamide
LEV: Levetiracetam
CZP: Clonazepam
VPA: Valproic acid
TPM: Topiramate
DZP: Diazepam
CLB: Clobazam
PPN: Perampanel
LTG: Lamotrigine
PHT: Phenytoin
PB: Phenobarbital
CBZ: Carbamazepine.
